# Efficient Synthesis and Antibacterial Evaluation of (±)-Yanglingmycin and Its Analogues

**DOI:** 10.3390/molecules21010096

**Published:** 2016-01-15

**Authors:** Wenjia Dan, Huiling Geng, Jianwen Qiao, Rui Guo, Shaopeng Wei, Longbo Li, Wenjun Wu, Jiwen Zhang

**Affiliations:** 1 College of Science, Northwest A & F University, Yangling 712100, Shaanxi, China; dwj586@gmail.com (W.D.); genghuiling5@163.com (H.G.); 18700427513@163.com (J.Q.); 18740456637@163.com (R.G.); lilongbo528@126.com (L.L.); 2 Key Laboratory of Botanical Pesticide R & D in Shaanxi Province, Northwest A & F University, Yangling 712100, Shaanxi, China; weishaopeng@nwsuaf.edu.cn (S.W.); wuwenjun@nwsuaf.edu.cn (W.W.)

**Keywords:** synthesis, Yanglingmycin, antibacterial activity, analogues

## Abstract

An efficient synthetic route was developed for the large-scale preparation of (±)-Yanglingmycin and its analogues. Three series of derivatives of (±)-Yanglingmycin were synthesized and the structures of all compounds were elucidated by analyses of NMR and ESI-MS spectra data. Moreover, their antibacterial activities against seven species of bacteria were systematically evaluated by the micro-broth dilution method, most of which displayed considerable activity. It was worth noting that compounds **5b**, **5c**, **5d**, **6g**, and **7** were found to be the most promising leading candidates, with peak MIC values of 0.98 μg·mL^−1^ for *Bacillus subtilis*, which is superior to positive controls (MIC = 3.91 μg·mL^−1^). The above results might lay the firm foundation for the design and synthesis of novel antibacterial drugs based on (±)-Yanglingmycin.

## 1. Introduction

**Figure 1:**
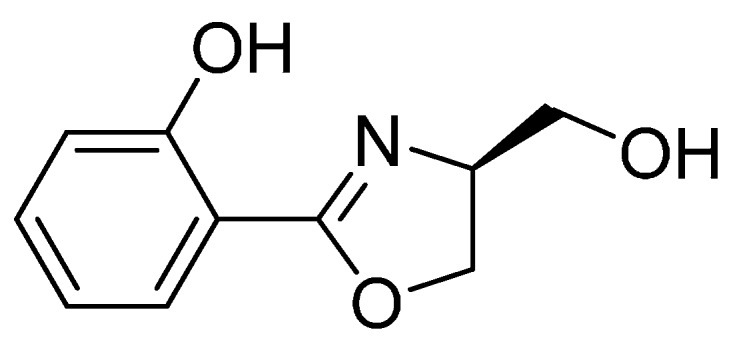
The structure of (−)-Yanglingmycin.

Since the 1940s, antibiotics have been widely used in daily life mainly due to their key role in prevention and control of human, animal, and plant diseases, *etc.* [1]. Since the effective targets of the antibiotics are still rather limited [2], the abuse of antibiotics resulted in the target sites becoming less sensitive. This context has led to the emergence of antibiotic resistance, for higher doses of antibiotics required to successfully cure certain bacterial infections, with some antibiotics losing their antibiotic activity completely [3,4]. In 2013, biologically-active natural product (−)-Yanglingmycin (Figure 1) was carefully isolated from the fermentation broth of *Streptomyces djakartensis* by our group [5]. As a 2-aryl-substituted 4,5-dihydrooxazol derivative, (−)-Yanglingmycin has been found to possess wide-range functionality and exhibited a variety of biological activities useful for applications, such as pharmaceutical drugs, polymeric materials, insecticides, and so on [6,7]. As reported by us recently, both (−)-Yanglingmycin and (+)-Yanglingmycin exhibited satisfying antibacterial activity for *Pseudomonas syringae* pv. *Actinidiae* and *Ralstonia solanacearum* with peak MIC values 7.81 and 15.62 μg·mL^−1^, respectively [8]*.* As far as the antibacterial data of Yanglingmycin was concerned, we postulated that this compound has the potential to act as the lead compound for the development of new antibacterial drugs. Unfortunately, the content of (−)-Yanglingmycin in *Streptomyces djakartensis* fermentation broth was less than 2 μg·mL^−1^, obviously, which could not provide enough pure target compound for our further study. In the last decades, the total synthesis of oxazole compounds has attracted more and more attention of researchers all over the world [9,10,11,12,13]. In order to obtain more substrates for drug development, an efficient, low-cost, and convenient route was developed to synthesize (±)-Yanglingmycin, based on our previous studies [5,8,14]. To our delight, (−)-Yanglingmycin showed considerable MIC values against all tested bacteria. Herein, a series of hydroxyl ester and hydroxyl ether derivatives of the racemic compound and its phenyl different-substituted analogues were designed and synthesized to further explore the antibacterial potency. Moreover, all compounds were successively screened against several Gram-negative and Gram-positive bacteria species, which consisted of *Bacillus cereus*, *Bacillus subtilis*, *Staphylococcus aureus*, *Escherichia coli*, *Pseudomonas syringae* pv. *Actinidiae*, *Pseudomonas solanacearum*, and *Ralstonia solanacearum*.

## 2. Results and Discussion

### 2.1. Preparation of 2-Arylthiazoline Analogues

**Scheme 1:**
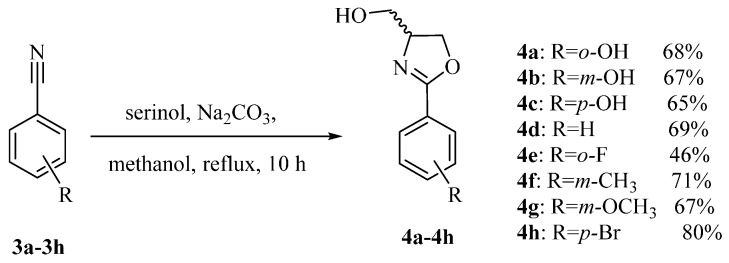
The synthetic route of (±)-Yanglingmycin and its analogues.

**Scheme 2:**
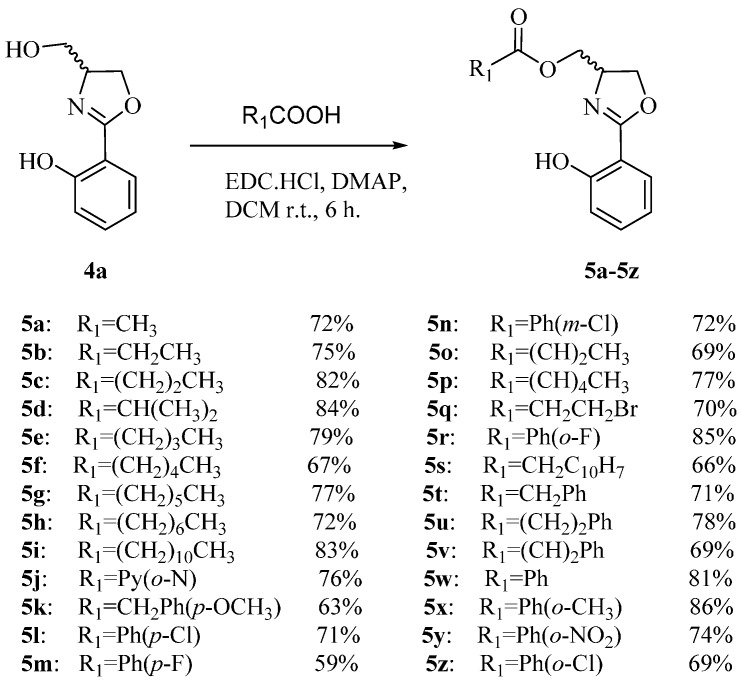
The synthetic route of esterified derivatives of (±)-Yanglingmycin.

**Scheme 3:**
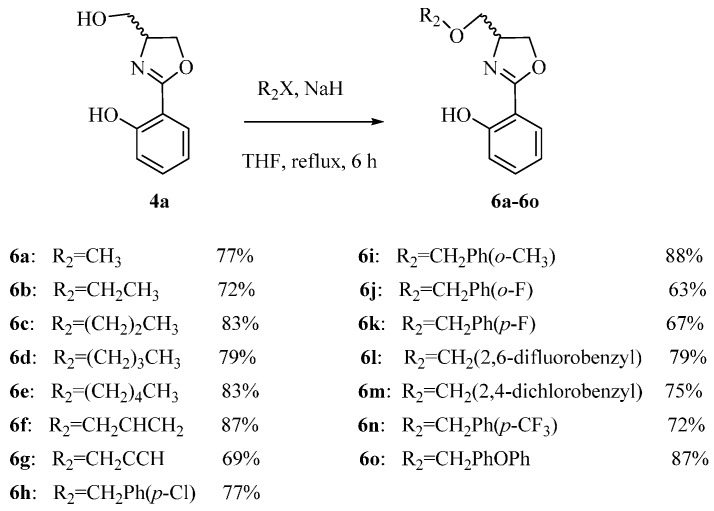
The synthetic route etherified derivatives of (±)-Yanglingmycin.

**Scheme 4:**
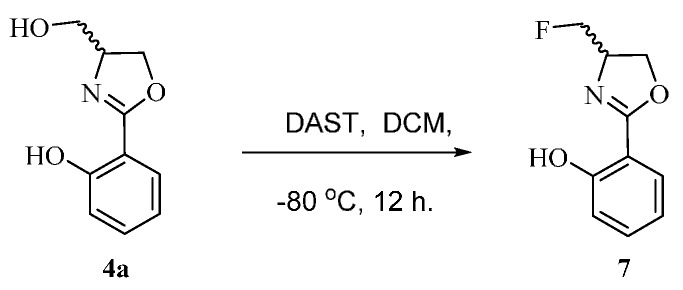
The synthetic route fluorinated derivatives of (±)-Yanglingmycin.

Large scale synthesis of (±)-Yanglingmycin and its analogues have been executed via an efficient pathway outlined in Scheme 1 [13]. In addition, series of derivatives of (±)-Yanglingmycin were prepared as well as assayed the antibacterial activities. Twenty-six derivatives **5a**–**5z** were afforded in 59%–86% yields by esterification reactions using catalytic amount of 4-*N*,*N*-dimethylaminepyridine and EDC·HCl (Scheme 2) [15]. Simultaneously, etherification of the alcoholic hydroxyl groups of compound **4a** provided fifteen derivatives **6a**–**6o** with yields 63%–88% (Scheme 3) [16,17]. Only one phenolic hydroxyl group usually reacted with an alkylating agent more easily than the alcoholic hydroxyl group under the base conditions. However, (±)-Yanglingmycin took a prior reaction on the alcoholic hydroxyl. From its molecular structure, we could infer that the activation energy of phenolic hydroxyl may be increased because of the presence of intramolecular hydrogen bonds. A fluorinated compound was afforded via (±)-Yanglingmycin reacted with DAST (Scheme 4) [18,19].

The preliminary results proved that compounds **5a**, **5b**, **5c**, **5d**, **5e**, **5f**, **5g**, **6f**, **6g**, and **7** were found to possess superior activity than (±)-Yanglingmycin. According to the evaluation results of MIC values, compounds **5b**, **5c**, **5d**, **6g**, and **7** have more potential to be the leading compounds for further medicine-relevant studies, compared to none of the **4b**–**h** MICs within 31.25 μg·mL^−1^. Taking a comprehensive view of the relationship between the compounds structure and its antibacterial data, most compounds lost their activity after the alcoholic hydroxyl derivatized or the 2-hydroxy substitution of the phenyl ring disappeared. However, short-chain ester group and electron-deficient ether moiety contribute significantly to the potency of antibacterial activity. Meanwhile, alcoholic hydroxyl fluorinate increased the antibacterial activity greatly.

### 2.2. Antibacterial Activities Assay

All the synthesized compounds were evaluated for their *in vitro* antibacterial activities against four Gram-negative (*Escherichia coli*, *Pseudomonas syringae* pv. *Actinidiae*, *Pseudomonas solanacearum*, *Ralstonia solanacearum*) and three Gram-positive bacteria (*Bacillus cereus*, *Bacillus subtilis*, *Staphylococcus aureus*). The minimum inhibitory concentrations (MICs) were determined by the double-dilution method using ampicillin sodium and fosfomycin sodium as the positive controls. All data of MIC values were summarized in Table 1 and Table 2.

**Table 1:** The MICs of (±)-Yanglingmycin and its derivatives.

Compound	*Bacillus cereus*	*Bacillus subtilis*	*Staphylococcus aureus*	*Escherichia coli*	*Pseudomonas syringae* pv. *actinidiae*	*Pseudomonas solanacearum*	*Ralstonia solanacearum*
(±)-Yanglingmycin	15.62 (±2.25)	15.62 (±2.25)	31.25 (±2.98)	7.81 (±1.13)	7.81 (±0.56)	15.62 (±2.25)	15.62 (±2.25)
**5a**	62.50 (±5.96)	7.81 (±1.13)	7.81 (±0.56)	>125 (±0)	31.25 (±1.13)	31.25 (±0)	7.81 (±1.13)
**5b**	15.62 (±0)	7.81 (±1.13)	7.81 (±0)	125 (±0)	31.25 (±0)	62.50 (±0.40)	0.98 (±0)
**5c**	3.91 (±0.56)	0.98 (±0)	3.91 (±0)	125 (±0)	31.25 (±1.13)	31.25 (±1.13)	1.95 (±0)
**5d**	7.81 (±1.13)	7.81 (±0)	15.62 (±1.13)	>125 (±0)	125 (±0)	125 (±2.25)	3.91 (±0.40)
**5e**	15.62 (±0)	15.62 (±0)	15.62 (±0)	>125 (±0)	125 (±1.13)	125 (±2.25)	31.25 (±1.13)
**5f**	31.25 (±2.25)	7.81 (±1.13)	31.25 (±1.13)	>125 (±0)	>125 (±0)	125 (±0)	3.91 (±0.56)
**5g**	62.50 (±1.13)	7.81 (±0)	31.25 (±0)	>125 (±0)	>125 (±0)	125 (±0)	15.62 (±0)
**5h**	62.50 (±0)	31.25 (±2.25)	62.50 (±0)	>125 (±0)	>125 (±0)	125 (±2.25)	31.25 (±0)
**5i**	125 (±1.13)	62.50 (±2.25)	62.50 (±1.13)	>125 (±0)	>125 (±0)	15.62 (±0)	62.50 (±1.13)
**5j**	15.62 (±2.25)	62.50 (±9.02)	62.50 (±2.25)	15.62 (±0.56)	125 (±1.13)	125 (±0)	31.25 (±2.98)
**5k**	62.50 (±0)	62.50 (±1.13)	62.50 (±0)	>125 (±0)	>125 (±0)	125 (±2.25)	62.50 (±0)
**5l**	62.50 (±0)	31.25 (±0)	62.50 (±0)	125 (±1.13)	>125 (±0)	62.50 (±0.53)	62.50 (±1.13)
**5m**	62.50 (±0)	31.25 (±0)	62.50 (±0)	125 (±0)	>125 (±0)	62.50 (±0)	62.50 (±0)
**5n**	62.50 (±5.96)	31.25 (±0)	62.50 (±2.25)	125 (±5.96)	>125 (±0)	62.50 (±0.56)	62.50 (±1.13)
**5o**	62.50 (±0)	62.50 (±0)	62.50 (±0)	>125 (±0)	>125 (±0)	125 (±2.25)	62.50 (±0)
**5p**	62.50 (±0)	31.25 (±1.13)	62.50 (±0)	125 (±0)	125 (±0)	125 (±0)	31.25 (±0.56)
**5q**	31.25 (±0)	62.50 (±0)	125 (±2.25)	>125 (±0)	>125 (±0)	125 (±1.13)	15.62 (±0)
**5r**	31.25 (±2.25)	15.62 (±0)	62.50 (±0)	125 (±0)	125 (±1.13)	15.62 (±0)	31.25 (±0)
**5s**	62.50 (±9.02)	31.25 (±0)	31.25 (±1.13)	62.50 (±1.13)	125 (±0)	62.50 (±2.25)	62.50 (±1.13)
**5t**	62.50 (±0)	62.50 (±0)	62.50 (±0)	125 (±0)	125 (±1.13)	125 (±1.13)	62.50 (±0)
**5u**	125 (±0)	62.50 (±0)	125 (±0)	>125 (±0)	125 (±0)	125 (±2.25)	125 (±0)
**5v**	62.50 (±5.96)	62.50 (±2.25)	125 (±0)	>125 (±0)	125 (±0)	125 (±0)	62.50 (±0)
**5w**	125 (±0)	62.50 (±1.13)	62.50 (±2.25)	62.50 (±1.13)	125 (±5.96)	62.50 (±1.13)	62.50 (±2.25)
**5x**	62.50 (±0)	31.25 (±1.13)	62.50 (±0)	62.50 (±0)	125 (±0)	62.50 (±2.25)	31.25 (±1.13)
**5y**	31.25 (±2.25)	31.25 (±0)	62.50 (±0)	125 (±0)	125 (±2.25)	62.50 (±0)	31.25 (±0)
**5z**	62.50 (±2.25)	62.50 (±0)	125 (±2.25)	>125 (±0)	>125 (±0)	62.50 (±1.13)	62.50 (±2.25)
Ampicillin Sodium	3.91 (±0)	7.81 (±1.13)	3.91 (±0.53)	1.95 (±0.40)	0.98 (±0)	7.81 (±0)	7.81 (±0.56)
Fosfomycin Sodium	3.91 (±0.53)	15.62 (±0)	3.91 (±0.53)	0.98 (±0)	>125 (±0)	125 (±0)	1.95 (±0.40)

**Table 2:** The MICs of (±)-Yanglingmycin and its derivatives.

Compound	*Bacillus cereus*	*Bacillus subtilis*	*Staphylococcus aureus*	*Escherichia coli*	*Pseudomonas syringae* pv. *actinidiae*	*Pseudomonas solanacearum*	*Ralstonia solanacearum*
(±)-Yanglingmycin	15.62 (±2.25)	15.62 (±2.25)	31.25 (±2.98)	7.81 (±1.13)	7.81 (±0.56)	15.62 (±2.25)	15.62 (±2.25)
**6a**	62.50 (±5.96)	31.25 (±4.06)	125 (±0)	125 (±0)	31.25 (±0)	31.25 (±4.51)	125 (±0)
**6b**	31.25 (±0)	15.62 (±2.25)	62.50 (±5.96)	31.25 (±0)	1.95 (±0)	15.62 (±2.25)	62.50 (±0)
**6c**	62.50 (±4.06)	31.25 (±0)	62.50 (±1.13)	125 (±0)	31.25 (±0.56)	31.25 (±0)	62.50 (±0)
**6d**	62.50 (±0)	31.25 (±2.25)	62.50 (±0)	62.50 (±1.13)	62.50 (±2.25)	15.62 (±0)	31.25 (±1.13)
**6e**	31.25 (±0)	62.50 (±0)	62.50 (±0)	62.50 (±1.13)	62.50 (±0)	31.25 (±0)	31.25 (±0)
**6f**	15.62 (±1.13)	7.81 (±0.53)	15.62 (±0.40)	125 (±0)	3.91 (±0)	7.81 (±0)	15.62 (±1.13)
**6g**	15.62 (±0)	15.62 (±0)	7.81 (±0)	125 (±0)	0.98 (±0)	0.98 (±0)	7.81 (±0)
**6h**	125 (±0)	62.50 (±0)	62.50 (±0)	>125 (±0)	125 (±0)	125 (±4.06)	125 (±4.51)
**6i**	62.50 (±0.53)	62.50 (±4.51)	62.50 (±1.13)	125 (±0)	62.50 (±2.25)	62.50 (±0)	125 (±0)
**6j**	31.25 (±0.40)	31.25 (±0)	31.25 (±0)	125 (±0)	62.50 (±0)	62.50 (±1.13)	62.50 (±0)
**6k**	62.50 (±0)	62.50 (±0.53)	62.50 (±0)	>125 (±0)	125 (±0)	125 (±2.98)	125 (±0)
**6l**	31.25 (±0)	31.25 (±0.40)	31.25 (±0.56)	125 (±0)	31.25 (±0)	15.62 (±0)	31.25 (±0)
**6m**	62.50 (±1.13)	31.25 (±0)	62.50 (±0)	125 (±0)	62.50 (±1.13)	62.50 (±0.53)	62.50 (±1.13)
**6n**	31.25 (±0)	31.25 (±0)	31.25 (±0)	125 (±0)	31.25 (±1.13)	31.25 (±0.40)	31.25 (±0)
**6o**	62.50 (±0)	31.25 (±0.53)	62.50 (±0)	>125 (±0)	62.50 (±2.25)	62.50 (±2.25)	31.25 (±0)
**7**	31.25 (±1.13)	15.62 (±0)	7.81 (±0)	31.25 (±0.56)	0.98 (±0)	0.98 (±0)	15.62 (±0.40)
Ampicillin Sodium	3.91 (±0)	7.81 (±1.13)	3.91 (±0.53)	1.95 (±0.40)	0.98 (±0)	7.81 (±0)	7.81 (±0.56)
Fosfomycin Sodium	3.91 (±0.53)	15.62 (±0)	3.91 (±0.53)	0.98 (±0)	>125 (±0)	125 (±0)	1.95 (±0.40)

## 3. Experimental Section

### 3.1. Chemistry

General unless otherwise noted, all reagents and solvents were purchased from commercial suppliers and used without further purification, which were of analytical reagent (AR) grade. TLC was performed on GF_254_ silica gel plates (Qingdao Haiyang Co., Ltd., Qingdao, Shandong, China). Column chromatography was carried out with silica gel (Qingdao Haiyang Co., Ltd.); all compounds were eluted with petroleum ether and ethyl acetate in sequence. Melting point (m.p.) was determined on a Yanagimoto apparatus (uncorrected). ^1^H-NMR and ^13^C-NMR spectra were performed on a Bruker-Avance-500 spectrometer (Bruker Daltonics Inc., Bremen, Germany) with DMSO-*d*_6_ or CDCl_3_ as solvent and SiMe_4_ (tetramethylsilane) as the internal standard. MS were recorded on an electrospray ionization (ESI) conditions by using a Thermo LCQ Fleet instrument (Thermo Fisher Scientific Co., Waltham, MA, USA). HR-MS were recorded on an Agilent 1290-6224 instrument (Agilent, Santa Clara, CA, USA).

#### 3.1.1. Synthesis of **4a**–**4h**

The solution of substituted benzonitrile (100 mmol), serinol (54.660 g, 600 mmol), and Na_2_CO_3_ (10.599 g, 100 mmol) in anhydrous methanol (100 mL) was heated to reflux for 10 h. After the reaction solution was cooled to room temperature, the solvent was removed under vacuum, the resulting residue was then diluted with anhydrous CH_2_Cl_2_ (30 mL). The organic fractions were successively washed with an aqueous saturated solution of NH_4_Cl (30 mL × 2), brine (30 mL × 3), and dried over anhydrous Na_2_SO_4_. The solvent was got rid of under vacuum to afford a scarlet residue which was purified by flash column chromatography using petroleum ether/ethyl acetate (8:1, *v*/*v*) as the eluent.

*2-(4-(Hydroxymethyl)-4,5-dihydrooxazol-2-yl)phenol* (**4a**) Yield: 68%–78%; white solids; m.p. 78–80 °C; ESI-MS *m/z* 194.07 [M + H]^+^; HR-MS *m/z*: Calcd for C_10_H_12_NO_3_: 194.0817 [M + H]^+^; found 194.0803 [M + H]^+^; ^1^H-NMR (CDCl_3_, 500 MHz) δ 7.66 (dd, *J* = 1.5, 7.5 Hz, 1H), 7.39 (m, 1H), 7.01 (d, *J* = 8.0 Hz, 1H), 6.89 (m, 1H), 4.50 (m, 2H), 4.36 (t, *J* = 6.0 Hz, 1H), 3.89 (dd, *J* = 3.5, 11.5 Hz, 1H), 3.71 (dd, *J* = 3.5, 11.5 Hz, 1H); ^13^C-NMR (125 MHz, CDCl_3_) δ 166.93, 159.78, 133.68, 128.22, 118.81, 116.75, 110.40, 68.58, 66.87, 63.95.

*3-(4-(Hydroxymethyl)-4,5-dihydrooxazol-2-yl)phenol* (**4b**) Yield: 67%; white solids; m.p. 91–93 °C; ESI-MS *m/z* 194.17 [M + H]^+^; ^1^H-NMR (500 MHz, CDCl_3_) δ 7.48 (m, 1H), 7.44 (m, 1H), 7.28 (m, 1H), 7.03 (m, 1H), 4.52 (dd, *J* = 3.5, 9.5 Hz, 1H), 4.42 (m, 1H), 4.36 (t, *J* = 7.5 Hz, 1H), 3.95 (dd, *J* = 3.5, 11.5 Hz, 1H), 3.67 (m, 1H); ^13^C-NMR (125 MHz, CDCl_3_) δ 165.41, 159.86, 128.98, 127.49, 120.81, 118.42, 113.19, 69.27, 68.11, 64.58.

*4-(4-(Hydroxymethyl)-4,5-dihydrooxazol-2-yl)phenol* (**4c**) Yield: 65%; white solids; m.p. 192–194 °C; ESI-MS *m/z* 194.02 [M + H]^+^; ^1^H-NMR (500 MHz, DMSO-*d*_6_) δ 10.03 (br.s., 1H), 7.69 (d, *J* = 8.8 Hz, 2H), 6.81 (d, *J* = 8.5 Hz, 2H), 4.79 (t, *J* = 5.5 Hz, 1H), 4.37 (t, *J* = 8.2 Hz, 1H), 4.19 (t, *J* = 6.0 Hz, 1H), 3.62 (m, 1H), 3.41 (td, *J* = 5.5, 10.8 Hz, 1H); ^13^C-NMR (125 MHz, DMSO-*d*_6_) δ 163.25, 160.66, 130.17, 118.3, 115.65, 69.95, 68.43, 63.74.

*(2-Phenyl-4,5-dihydrooxazol-4-yl)methanol* (**4d**) Yield: 69%; white solids; m.p. 82–84 °C; ESI-MS *m/z* 178.12 [M + H]^+^; ^1^H-NMR (500 MHz, CDCl_3_) δ 7.98 (m, 2H), 7.47 (m, 1H), 7.38 (m, 2H), 4.43 (m, 1H), 4.34 (m, 1H), 4.49 (m, 1H), 3.98 (dd, *J* = 3.5, 11.5 Hz, 1H), 3.67 (dd, *J* = 4.0, 11.5 Hz, 1H); ^13^C-NMR (125 MHz, CDCl_3_) δ 165.61, 131.57, 128.37, 128.31, 127.21, 69.29, 68.08, 63.94.

*(2-(2-Fluorophenyl)-4,5-dihydrooxazol-4-yl)methanol* (**4e**) Yield: 46%; yellow oil; ESI-MS *m/z* 196.16 [M + H]^+^; ^1^H-NMR (500 MHz, CDCl_3_) δ 7.88 (td, *J* = 1.5, 7.5 Hz, 1H), 7.47 (m, 1H), 7.19 (m, 2H), 4.50 (m, 2H), 4.34 (m, 1H), 3.85 (dd, *J* = 3.0, 11.5 Hz, 1H), 3.71 (dd, *J* = 4.0, 11.5 Hz, 1H); ^13^C-NMR (125 MHz, CDCl_3_) δ 162.28, 162.21, 162.17, 160.22, 133.16, 133.09, 131.19, 124.01, 123.98, 116.80, 116.63, 69.06, 68.42, 64.16.

*(2-(m-Tolyl)-4,5-dihydrooxazol-4-yl)methanol* (**4f**) Yield: 71%; white solids; m.p. 146–148 °C; ESI-MS *m/z* 192.12 [M + H]^+^; ^1^H-NMR (500 MHz, CDCl_3_) δ 7.71 (s, 1H), 7.68 (m, 2H), 7.28 (m, 2H), 4.49 (dd, *J* = 7.5, 9.5 Hz, 1H), 4.44 (m, 1H), 4.34 (m, 1H), 3.96 (dd, *J* = 3.5, 11.5 Hz, 1H), 3.68 (dd, *J* = 4.0, 11.5 Hz, 1H), 2.36 (s, 3H); ^13^C-NMR (125 MHz, CDCl_3_) δ 165.73, 138.05, 132.31, 128.92, 128.23, 127.16, 125.44, 69.26, 68.07, 64.06, 21.23.

*(2-(3-Methoxyphenyl)-4,5-dihydrooxazol-4-yl)methanol* (**4g**) Yield: 67%; white solids; m.p. 123–125 °C; ESI-MS *m/z* 208.03 [M + H]^+^; ^1^H-NMR (500 MHz, CDCl_3_) δ 7.49 (m, 1H), 7.42 (m, 1H), 7.29 (m, 1H), 7.02 (m, 1H), 4.50 (dd, *J* = 3.5, 9.5 Hz, 1H), 4.44 (m, 1H), 4.35 (t, *J* = 7.5 Hz, 1H), 3.97 (dd, *J* = 3.5, 11.5 Hz, 1H), 3.83 (s, 3H), 3.68 (m, 1H); ^13^C-NMR (125 MHz, CDCl_3_) δ 165.47, 159.46, 129.38, 128.53, 120.83, 118.34, 112.69, 69.34, 68.12, 64.03, 55.40.

*(2-(4-Bromophenyl)-4,5-dihydrooxazol-4-yl)methanol* (**4h**) Yield: 80%; yellow oil; ESI-MS *m/z* 255.01:257.13 (1:1), [M + H]^+^; ^1^H-NMR (500 MHz, CDCl_3_) δ 7.74 (m, 2H), 7.51 (m, 2H), 4.51 (dd, *J* = 7.5, 9.5 Hz, 1H), 4.41 (m, 1H), 4.36 (m, 1H), 3.98 (dd, *J* = 3.5, 11.5 Hz, 1H), 3.68 (dd, *J* = 4.0, 11.5 Hz, 1H); ^13^C-NMR (125 MHz, CDCl_3_) δ 175.12, 164.74, 131.59, 129.85, 126.30, 126.21, 69.44, 68.19, 63.85.

#### 3.1.2. Synthesis of **5a**–**5z**

A solution of compound **4a** (193 mg, 1.0 mmol), carboxylic acid (1.2 mmol), EDC·HCl (288 mg, 1.5 mmol), and DMAP (6 mg, 5%) in anhydrous CH_2_Cl_2_ (8 mL) is stirred at room temperature for 6 h. Then, the reaction solution was quenched with saturated aqueous NaHCO_3_ (50 mL), concentrated under reduced pressure until approximately 30 mL remained. The organic layer was separated with CH_2_Cl_2_ (30 mL × 3) and washed sequentially with water (30 mL × 2), brine (30 mL × 3), and dried over anhydrous Na_2_SO_4_. The solvent is concentrated under reduced pressure, and purified by flash column chromatography using petroleum ether/ethyl acetate (10:1, *v*/*v*) as the eluent.

*(2-(2-Hydroxyphenyl)-4,5-dihydrooxazol-4-yl)methyl acetate* (**5a**) Yield: 72%; yellow oil; ESI-MS *m/z* 236.12 [M + H]^+^; ^1^H-NMR (500 MHz, CDCl_3_) δ 7.66 (dd, *J* = 2.0, 9.0 Hz, 1H), 7.41 (m, 1H), 7.02 (dd, *J* = 1.0, 8.5 Hz, 1H), 6.90 (m, 1H), 4.65 (m, 1H), 4.51 (dd, *J* = 8.5, 9.5 Hz, 1H), 4.31 (m, 2H), 4.21 (dd, *J* = 5.5, 21.5 Hz, 1H), 2.07 (s, 3H); ^13^C-NMR (125 MHz, CDCl_3_) δ 170.83, 166.74, 159.95, 133.75, 128.18, 118.72, 116.88, 110.29, 69.14, 65.43, 64.21, 20.78.

*(2-(2-Hydroxyphenyl)-4,5-dihydrooxazol-4-yl)methyl propionate* (**5b**) Yield: 75%; orange oil; ESI-MS *m/z* 250.17 [M + H]^+^; ^1^H-NMR (500 MHz, CDCl_3_) δ 7.66 (dd, *J* = 1.5, 8.0 Hz, 1H), 7.40(m, 1H), 7.02(dd, *J* = 1.0, 7.5 Hz, 1H), 6.89 (m, 1H), 4.65 (m, 1H), 4.51 (dd, *J* = 1.5, 8.0 Hz, 1H), 4.32 (m, 2H), 4.23 (dd, *J* = 5.5, 11.0 Hz, 1H), 2.34 (q, *J* = 7.5 Hz, 2H), 1.12 (m, 3H); ^13^C-NMR (125 MHz, CDCl_3_) δ 174.25, 166.71, 159.95, 133.72, 128.16, 118.71, 116.87, 110.31, 69.13, 65.25, 64.27, 27.44, 9.05.

*(2-(2-Hydroxyphenyl)-4,5-dihydrooxazol-4-yl)methyl butyrate* (**5c**) Yield: 82%; yellow oil; ESI-MS *m/z* 264.09 [M + H]^+^; ^1^H-NMR (500 MHz, CDCl_3_) δ 11.88 (s, 1H, Ar-OH), 7.66 (dd, *J* = 2.0, 8.0 Hz, 1H), 7.40 (m, 1H), 7.02 (dd, *J* = 1.0, 8.5 Hz, 1H), 6.89 (m, 1H), 4.65 (m, 1H), 4.50 (dd, *J* = 9.0, 10.0 Hz, 1H), 4.31 (m, 2H), 4.24 (dd, *J* = 4.5, 9.5 Hz, 1H), 2.29 (t, *J* = 7.0 Hz, 2H), 1.66 (m, 2H), 0.91 (t, *J* = 7.5 Hz, 3H); ^13^C-NMR (125 MHz, CDCl_3_) δ 173.47, 166.71, 159.96, 133.74, 128.17, 118.73, 116.87, 110.32, 69.13, 65.25, 64.27, 36.13, 18.45, 13.60.

*(2-(2-Hydroxyphenyl)-4,5-dihydrooxazol-4-yl)methyl isobutyrate* (**5d**) Yield: 84%; yellow oil; ESI-MS *m/z* 264.13 [M + H]^+^; ^1^H-NMR (500 MHz, CDCl_3_) δ 7.65 (dd, *J* = 2.0, 8.0 Hz, 1H), 7.40 (m, 1H), 7.01(dd, *J* = 0.5, 8.5 Hz, 1H), 6.89 (m, 1H), 4.65 (m, 1H), 4.50 (m, 1H), 4.30 (m, 1H), 4.27 (m, 1H), 2.59 (m, 1H), 1.13 (t, *J* = 8.0 Hz, 6H); ^13^C-NMR (125 MHz, CDCl_3_) δ 176.85, 166.70, 159.95, 133.71, 128.14, 118.70, 116.86, 110.30, 69.06, 65.08, 64.32, 33.93, 18.91, 18.87, 1.03.

*(2-(2-Hydroxyphenyl)-4,5-dihydrooxazol-4-yl)methyl pentanoate* (**5e**) Yield: 79%; orange oil; ESI-MS *m/z* 278.09 [M + H]^+^; ^1^H-NMR (500 MHz, CDCl_3_) δ 11.60 (br. s, 1H, Ar-OH), 7.65 (d, *J* = 7.5 Hz, 1H), 7.39 (t, *J* = 7.5 Hz, 1H), 7.02 (d, *J* = 8.0 Hz, 1H), 6.88 (t, *J* = 7.0 Hz, 1H), 4.62 (m, 1H), 4.48 (t, *J* = 9.0 Hz, 1H), 4.27 (m, 3H), 2.32 (m, 2H), 1.57 (m, 2H), 1.32 (m, 2H), 0.86 (t, *J* = 6.5 Hz, 3H); ^13^C-NMR (125 MHz, CDCl_3_) δ 173.66, 166.70, 159.94, 133.71, 128.15, 118.71, 116.86, 110.30, 69.09, 65.14, 64.27, 33.88, 26.98, 22.19, 13.63.

*(2-(2-Hydroxyphenyl)-4,5-dihydrooxazol-4-yl)methyl hexanoate* (**5f**) Yield: 67%; orange oil; ESI-MS *m/z* 292.17 [M + H]^+^; ^1^H-NMR (500 MHz, CDCl_3_) δ 11.88(br. s, 1H, Ar-OH), 7.66 (dd, *J* = 2.0, 8.0 Hz, 1H), 7.40 (m, 1H), 7.02 (dd, *J* = 1.0, 8.5 Hz, 1H), 6.89 (m, 1H), 4.63 (m, 1H), 4.50 (dd, *J* = 9.0, 10.0 Hz, 1H), 4.30 (m, 3H), 2.30 (t, *J* = 7.5 Hz, 2H), 1.61 (m, 2H), 1.29(m, 4H), 0.85 (t, *J* = 7.0 Hz, 3H); ^13^C-NMR (125 MHz, CDCl_3_) δ 173.67, 166.70, 159.94, 133.70, 128.15, 118.71, 116.86, 110.32, 69.10, 65.14, 64.29, 34.14, 31.22, 24.60, 22.25, 13.84.

*(2-(2-Hydroxyphenyl)-4,5-dihydrooxazol-4-yl)methyl heptanoate* (**5g**) Yield: 77%; pale yellow oil; ESI-MS *m/z* 306.13 [M + H]^+^; ^1^H-NMR (500 MHz, CDCl_3_) δ 11.87 (br. s, 1H, Ar-OH), 7.66 (d, *J* = 7.0 Hz, 1H), 7.38 (t, *J* = 6.5 Hz, 1H), 7.02 (d, *J* = 9.0 Hz, 1H), 6.88 (t, *J* = 6.5 Hz, 1H), 4.61 (m, 1H), 4.48 (t, *J* = 8.0 Hz, 1H), 4.28 (m, 3H), 2.30 (t, *J* = 6.5 Hz, 2H), 1.57 (t, *J* = 6.5 Hz, 2H), 1.25 (m, 6H), 0.86 (t, *J* = 6.5 Hz, 3H); ^13^C-NMR (125 MHz, CDCl_3_) δ 173.65, 166.70, 159.95, 133.71, 128.15, 118.70, 116.86, 110.30, 69.10, 65.12, 64.28, 34.18, 31.39, 28.75, 24.88, 22.42, 14.01.

*(2-(2-Hydroxyphenyl)-4,5-dihydrooxazol-4-yl)methyl octanoate* (**5h**) Yield: 72%; yellow oil; ESI-MS *m/z* 320.17 [M + H]^+^; ^1^H-NMR (500 MHz, CDCl_3_) δ 11.84 (br. s, 1H, Ar-OH), 7.66 (dd, *J* = 1.5, 9.0 Hz, 1H), 7.40 (m, 1H), 7.02 (dd, *J* = 1.0, 8.5 Hz, 1H), 6.89 (m, 1H), 4.64 (m, 1H), 4.50 (dd, *J* = 8.5, 9.5 Hz, 1H), 4.30 (m, 3H), 2.30 (t, *J* = 6.5 Hz, 2H), 1.60 (m, 2H), 1.29 (m, 8H), 0.87 (t, *J* = 7.0 Hz, 3H); ^13^C-NMR (125 MHz, CDCl_3_) δ 173.65, 166.69, 159.94, 133.71, 128.15, 118.70, 116.86, 110.31, 69.10, 65.12, 64.28, 34.18, 31.60, 29.04, 28.87, 24.92, 22.59, 14.06.

*(2-(2-Hydroxyphenyl)-4,5-dihydrooxazol-4-yl)methyl dodecanoate* (**5i**) Yield: 83%; pale yellow oil; ESI-MS *m/z* 376.22 [M + H]^+^; ^1^H-NMR (500 MHz, CDCl_3_) δ 7.66 (dd, *J* = 1.5, 9.0 Hz, 1H), 7.40 (m, 1H), 7.02 (dd, *J* = 0.5, 8.5 Hz, 1H), 6.89 (m, 1H), 4.64 (m, 1H), 4.50 (dd, *J* = 8.5, 9.5 Hz, 1H), 4.30 (m, 3H), 2.30 (t, *J* = 6.5 Hz, 2H), 1.60 (m, 2H), 1.31 (m, 16H), 0.88 (t, *J* = 7.0 Hz, 3H); ^13^C-NMR (125 MHz, CDCl_3_) δ 173.64, 166.71, 159.97, 133.71, 128.16, 118.70, 116.88, 110.31, 69.11, 65.12, 64.29, 34.18, 31.92, 29.60, 29.42, 29.34, 29.22, 29.09, 24.93, 22.69, 14.12.

*(2-(2-Hydroxyphenyl)-4,5-dihydrooxazol-4-yl)methyl isonicotinate* (**5j**) Yield: 76%; white solid; m.p. 111 °C; ESI-MS *m/z* 299.12 [M + H]^+^; ^1^H-NMR (500 MHz, CDCl_3_) δ 11.83 (br. s, 1H, Ar-OH), 8.76 (dd, *J =* 1.5, 5.0 Hz, 2H), 7.79 (dd, *J* = 1.5, 4.5 Hz, 2H), 7.69 (dd, *J* = 1.5, 8.0 Hz, 1H), 7.42 (m, 1H), 7.02 (dd, *J* = 0.5, 8.5 Hz, 1H), 6.91 (m, 1H), 4.80 (m, 1H), 4.59 (m, 3H), 4.39 (dd, *J* = 6.5, 8.5 Hz, 1H); ^13^C-NMR (125 MHz, CDCl_3_) δ 167.02, 164.89, 159.95, 150.68, 136.79, 133.93, 128.18, 122.83, 118.85, 116.93, 110.12, 68.98, 66.59, 64.18.

*(2-(2-Hydroxyphenyl)-4,5-dihydrooxazol-4-yl)methyl 2-(4-methoxyphenyl)acetate* (**5k**) Yield: 63%; colorless solid; m.p. 105–106 °C; ESI-MS *m/z* 342.21 [M + H]^+^; ^1^H-NMR (500 MHz, CDCl_3_) δ 7.64 (dd, *J* = 1.5, 8.0 Hz, 1H), 7.41 (m, 1H), 7.14 (m, 2H), 7.03 (dd, *J* = 1.0, 8.5 Hz, 1H), 6.90 (m, 1H), 6.78 (m, 2H), 4.61 (m, 1H), 4.43 (dd, *J* = 8.5, 9.5 Hz, 1H), 4.27 (dd, *J* = 3.0, 4.5 Hz, 2H), 4.19 (dd, *J* = 7.5, 8.5 Hz, 1H), 3.76 (s, 3H), 3.55 (s, 2H); ^13^C-NMR (125 MHz, CDCl_3_) δ 171.63, 166.70, 159.95, 158.72, 133.70, 130.17, 128.18, 125.62, 118.69, 116.85, 114.00, 110.30, 68.87, 65.43, 64.20, 55.22, 40.39.

*(2-(2-Hydroxyphenyl)-4,5-dihydrooxazol-4-yl)methyl 4-chlorobenzoate* (**5l**) Yield: 71%; white solid; m.p. 106 °C; ESI-MS *m/z* 332.06:334.11 (3:1), [M + H]^+^; ^1^H-NMR (500 MHz, CDCl_3_) δ 7.93 (m, 2H), 7.68 (dd, *J* = 1.5, 7.5 Hz, 1H), 7.42 (m, 3H), 7.02 (dd, *J* = 0.5, 8.0 Hz, 1H), 6.91 (m, 1H), 4.78 (m, 1H), 4.57 (m, 2H), 4.47 (dd, *J* = 4.5, 9.5 Hz, 1H), 4.39 (dd, *J* = 7.0, 8.5 Hz, 1H); ^13^C-NMR (125 MHz, CDCl_3_) δ 166.90, 165.48, 159.96, 139.78, 133.84, 131.05, 128.84, 128.16, 128.04, 118.80, 116.91, 110.21, 69.12, 66.11, 64.29.

*(2-(2-Hydroxyphenyl)-4,5-dihydrooxazol-4-yl)methyl 4-fluorobenzoate* (**5m**) Yield: 59%; white solid; m.p. 85–87 °C; ESI-MS *m/z* 316.12 [M + H]^+^; ^1^H-NMR (500 MHz, CDCl_3_) δ 8.02 (m, 2H), 7.68 (dd, *J* = 1.5, 8.0 Hz, 1H), 7.41 (m, 1H), 7.10 (m, 2H), 7.03 (dd, *J* = 1.0, 8.5 Hz, 1H), 6.91 (m, 1H), 4.78 (m, 1H), 4.57 (m, 2H), 4.47 (dd, *J* = 4.5, 9.5 Hz, 1H), 4.39 (dd, *J* = 7.0, 9.0 Hz, 1H); ^13^C-NMR (125 MHz, CDCl_3_) δ 166.95, 166.88, 165.36, 164.93, 159.98, 133.82, 132.29, 132.21, 128.17, 125.85, 118.78, 116.91, 115.75, 115.58, 110.24, 69.14, 66.02, 64.33.

**Figure 2:**
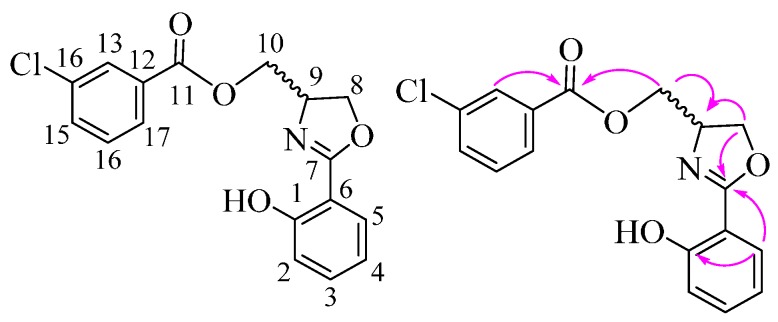
The main HMBC of compound **5n**.

*(2-(2-Hydroxyphenyl)-4,5-dihydrooxazol-4-yl)methyl 3-chlorobenzoate* (**5n**) Yield: 72%; white solid; m.p. 87–89 °C; ESI-MS *m/z* 332.11:333.06 (3:1), [M + H]^+^; ^1^H-NMR (500 MHz, CDCl_3_) δ 7.79 (m, 2H), 7.70 (dd, *J* = 2.0, 8.0 Hz, 1H), 7.40 (m, 1H), 7.36 (m, 1H), 7.30 (m, 1H), 7.01 (dd, *J* = 0.5, 8.5 Hz, 1H), 6.90 (m, 1H), 4.78 (m, 1H), 4.56 (m, 2H), 4.44 (m, 2H); ^13^C-NMR (125 MHz, CDCl_3_) δ 166.81, 166.45, 160.29, 138.20, 134.10, 133.72, 130.22, 129.45, 128.37, 128.27, 126.86, 118.83, 116.39, 110.38, 69.27, 65.69, 64.54. The structure of **5n** was also confirmed by 2D NMR data and can be seen in Table 3 and Figure 2.

**Table 3:** HMBC data of compound **5n**.

^13^C-NMR			^1^H-NMR		
C No.	δ_C_	HMBC Correlations	H No.	δ_H_	HMBC Correlations
1-C	159.94, C	2-H, 3-H, 5-H	Ar-OH	-	-
2-C	116.89, CH	3-H, 4-H	2-H	7.02, dd, *J* = 0.5, 9.0 Hz, 1H	1-C, 4-C, 6-C, 7-C
3-C	133.83, CH	4-H, 5-H	3-H	7.41–7.38, m, 1H	1-C, 2-C, 5-C
4-C	118.81, CH	2-H	4-H	6.90–6.87, m, 1H	2-C, 3-C, 5-C, 6-C
5-C	128.20, CH	3-H, 4-H	5-H	7.69, dd, *J* = 1.5, 8.0 Hz, 1H	1-C, 3-C, 7-C
6-C	110.21, C	2-H, 4-H	6-H	-	-
7-C	166.95, C	2-H, 4-H, 5-H, 8-H, 9-H	7-H	-	-
8-C	66.33, CH_2_	9-H, 10-H	8-H	4.57–4.54, m, 1H; 4.39, dd, *J* = 7.0, 9.0 Hz, 1H	7-C, 9-C, 10-C
9-C	64.26, CH	8-H, 10-H	9-H	4.78–4.73, m, 1H;	7-C, 8-C
10-C	69.19, CH_2_	8-H	10-H	4.54–4.52, m, 1H; 4.47, dd, *J* = 5.5, 11.5 Hz, 1H	8-C, 9-C, 11-C
11-C	165.13, C	10-H, 13-H, 16-H, 17-H	11-H	-	-
12-C	134.64, C	13-H, 16-H, 17-H	12-H	-	-
13-C	129.74, CH	15-H, 16-H, 17-H	13-H	7.95, t, *J* = 1.5 Hz, 1H	11-C, 12-C, 14-C, 15-C, 17-C
14-C	131.33, C	13-H, 15-H, 16-H	14-H	-	-
15-C	133.29, CH	13-H, 16-H, 17-H	15-H	7.53, dq, *J* = 1.5, 8.5 Hz, 1H	13-C, 14-C, 16-C, 17-C
16-C	129.80, CH	15-H, 17-H	16-H	7.37, t, *J* = 8.0 Hz, 1H	11-C, 12-C, 14-C, 15-C, 17-C
17-C	127.77, CH	13-H, 15-H, 16-H	17-H	7.87, dt, *J* = 1.5, 7.5 Hz, 1H	11-C, 12-C, 13-C, 15-C, 16-C

*(E)-(2-(2-Hydroxyphenyl)-4,5-dihydrooxazol-4-yl)methyl but-2-enoate* (**5o**) Yield: 69%; pale yellow solid; m.p. 106 °C; ESI-MS *m/z* 262.08 [M + H]^+^; ^1^H-NMR (500 MHz, CDCl_3_) δ 11.89 (br. s, 1H, Ar-OH), 7.66 (dd, *J* = 1.5, 7.5 Hz, 1H), 7.40 (m, 1H), 7.02 (m, 2H), 6.89 (m, 1H), 5.86 (dq, *J* = 1.5, 15.5 Hz, 1H), 4.67 (m, 1H), 4.51 (dd, *J* = 9.0, 10.0 Hz, 1H), 4.38 (dd, *J* = 4.5, 11.0 Hz, 1H), 4.30 (dd, *J* = 7.0, 8.5 Hz, 1H), 4.26 (dd, *J* = 4.5, 9.5 Hz, 1H), 1.87 (dd, *J* = 2.0, 7.0 Hz, 3H). ^13^C-NMR (125 MHz, CDCl_3_) δ 166.69, 166.22, 159.95, 145.77, 133.69, 128.16, 122.02, 118.70, 116.86, 110.34, 69.18, 65.14, 64.32, 18.04.

*(2E,4E)-(2-(2-Hydroxyphenyl)-4,5-dihydrooxazol-4-yl)methyl hexa-2,4-dienoate* (**5p**) Yield: 77%; dark green solid; m.p. 67–69 °C; ESI-MS *m/z* 288.10 [M + H]^+^; ^1^H-NMR (500 MHz, CDCl_3_) δ 11.90 (br. s, 1H, Ar-OH), 7.67 (dd, *J* = 1.5, 8.0 Hz, 1H), 7.40 (m, 1H), 7.26 (dd, *J* = 10.5, 15.5 Hz, 1H), 7.02 (dd, *J* = 1.0, 8.5 Hz, 1H), 6.89 (m, 1H), 6.20 (m, 2H), 5.77 (d, *J* = 15.5 Hz, 1H), 4.68 (m, 1H), 4.52 (m, 1H), 4.40 (m, 1H), 4.30 (dd, *J* = 7.0, 8.5 Hz, 1H), 4.26 (dd, *J* = 4.5, 9.5 Hz, 1H), 1.85 (m, 3H). ^13^C-NMR (125 MHz, CDCl_3_) δ 166.99, 166.70, 159.97, 145.97, 140.13, 133.68, 129.65, 128.16, 118.68, 118.03, 116.87, 110.35, 69.25, 65.24, 64.35, 18.67.

*(2-(2-Hydroxyphenyl)-4,5-dihydrooxazol-4-yl)methyl 3-bromopropanoate* (**5q**) Yield: 70%; pale yellow solid; m.p. 113–114 °C; ESI-MS *m/z* 328.17:330.21 (1:1), [M + H]^+^; ^1^H-NMR (500 MHz, CDCl_3_) δ 7.65 (dd, *J* = 1.5, 8.0 Hz, 1H), 7.39 (m, 1H), 7.00 (dd, *J* = 1.0, 7.5 Hz, 1H), 6.89 (m, 1H), 4.67 (m, 1H), 4.56 (dd, *J* = 1.5, 8.0 Hz, 1H), 4.32 (m, 2H), 4.20 (dd, *J* = 5.5, 11.0 Hz, 1H), 3.66 (t, 2H), 2.38 (q, *J* = 7.5 Hz, 2H); ^13^C-NMR (125 MHz, CDCl_3_) δ 174.31, 166.60, 159.88, 133.71, 128.16, 118.71, 116.57, 110.19, 69.03, 65.77, 64.37, 37.49, 27.01.

*(2-(2-Hydroxyphenyl)-4,5-dihydrooxazol-4-yl)methyl 2-fluorobenzoate* (**5r**) Yield: 85%; white solid; m.p. 83–84 °C; ESI-MS *m/z* 316.07 [M + H]^+^; ^1^H-NMR (500 MHz, CDCl_3_) δ 7.79 (dd, *J* = 1.5, 8.0 Hz, 1H), 7.67 (dd, *J* = 1.5, 8.0 Hz, 1H), 7.42 (m, 3H), 7.29 (m, 1H), 7.02 (dd, *J* = 0.5, 8.0 Hz, 1H), 6.90 (m, 1H), 4.79 (m, 1H), 4.57 (m, 2H), 4.49 (dd, *J* = 4.5, 9.5 Hz, 1H), 4.42 (dd, *J* = 7.0, 8.5 Hz, 1H); ^13^C-NMR (125 MHz, CDCl_3_) δ 166.92, 165.59, 159.94, 133.79, 133.77, 132.86, 131.67, 131.16, 129.57, 128.18, 126.67, 118.75, 116.88, 110.32, 69.17, 66.37, 64.20.

*(2-(2-Hydroxyphenyl)-4,5-dihydrooxazol-4-yl)methyl 2-(naphthalen-2-yl)acetate* (**5s**) Yield: 66%; white solid; m.p. 80 °C; ESI-MS *m/z* 362.17 [M + H]^+^; ^1^H-NMR (500 MHz, CDCl_3_) δ 7.94 (m, 1H), 7.83 (dd, *J* = 2.5, 7.5 Hz, 1H), 7.75 (m, 1H), 7.57 (dd, *J* = 2.0, 8.0 Hz, 1H), 7.48 (m, 2H), 7.39 (m, 1H), 7.34 (m, 2H), 7.01 (dd, *J* = 0.5, 8.0 Hz, 1H), 6.89 (m, 1H), 4.53 (m, 1H), 4.29 (dd, *J* = 4.0, 11.0 Hz, 1H), 4.24 (m, 2H), 4.06 (s, 2H), 3.98 (dd, *J* = 7.5, 9.0 Hz, 1H); ^13^C-NMR (125 MHz, CDCl_3_) δ 171.30, 166.63, 159.91, 133.75, 133.67, 131.91, 130.09, 128.75, 128.23, 128.16, 127.99, 126.40, 125.79, 125.42, 123.51, 118.64, 116.83, 110.22, 68.68, 65.46, 64.07, 39.05.

*(2-(2-Hydroxyphenyl)-4,5-dihydrooxazol-4-yl)methyl 2-phenylacetate* (**5t**) Yield: 71%; white solid; m.p. 106 °C; ESI-MS *m/z* 312.07 [M + H]^+^; ^1^H-NMR (500 MHz, CDCl_3_) δ 7.64 (dd, *J* = 1.5, 7.5 Hz, 1H), 7.41 (m, 1H), 7.25 (m, 5H), 7.02 (dd, *J* = 0.5, 8.0 Hz, 1H), 6.90 (m, 1H), 4.61 (m, 1H), 4.42 (dd, *J* = 8.5, 9.5 Hz, 1H), 4.30 (m, 2H), 4.18 (dd, *J* = 7.5, 9.0 Hz, 1H), 3.61 (s, 2H); ^13^C-NMR (125 MHz, CDCl_3_) δ 171.31, 166.70, 159.95, 133.72, 133.57, 129.13, 128.57, 128.21, 127.17, 118.69, 116.86, 110.29, 68.84, 65.50, 64.17, 41.30.

*(2-(2-Hydroxyphenyl)-4,5-dihydrooxazol-4-yl)methyl 3-phenylpropanoate* (**5u**) Yield: 78%; pale yellow oil; ESI-MS *m/z* 326.07 [M + H]^+^; ^1^H-NMR (500 MHz, CDCl_3_) δ 7.64 (dd, *J* = 1.5, 7.5 Hz, 1H), 7.41 (m, 1H), 7.24 (m, 5H), 7.02 (dd, *J* = 0.5, 8.0 Hz, 1H), 6.92 (m, 1H), 4.60 (m, 1H), 4.42 (dd, *J* = 8.5, 9.5 Hz, 1H), 4.30 (m, 2H), 4.04 (dd, *J* = 7.5, 9.0 Hz, 1H), 3.61 (t, *J* = 7.5 Hz, 2H), 2.83 (t, *J* = 7.5 Hz, 2H); ^13^C-NMR (125 MHz, CDCl_3_) δ 171.31, 166.70, 159.95, 133.72, 133.57, 129.13, 128.57, 128.21, 127.17, 118.69, 116.86, 110.29, 68.84, 65.50, 64.17, 41.30, 34.12.

*(2-(2-Hydroxyphenyl)-4,5-dihydrooxazol-4-yl)methyl cinnamate* (**5v**) Yield: 69%; white solid; m.p. 85 °C; ESI-MS *m/z* 324.22 [M + H]^+^; ^1^H-NMR (500 MHz, CDCl_3_) δ 7.65 (dd, *J* = 1.5, 7.5 Hz, 1H), 7.40 (m, 1H), 7.28 (m, 2H), 7.21 (m, 3H), 7.02 (dd, *J* = 0.5, 8.0 Hz, 1H), 6.89 (m, 1H), 4.58 (m, 1H), 4.41 (dd, *J* = 9.0, 10.0 Hz, 1H), 4.30 (dd, *J* = 4.5, 11.5 Hz, 1H), 4.20 (dd, *J* = 4.5, 9.5 Hz, 1H), 4.16 (dd, *J* = 7.0, 8.5 Hz, 1H), 2.92 (t, *J* = 7.5 Hz, 2H), 2.65 (t, *J* = 7.5 Hz, 2H); ^13^C-NMR (125 MHz, CDCl_3_) δ 172.65, 166.73, 159.95, 140.19, 133.74, 128.52, 128.23, 128.17, 126.35, 118.72, 116.88, 110.28, 69.08, 65.35, 64.17, 35.62, 30.86.

*(2-(2-Hydroxyphenyl)-4,5-dihydrooxazol-4-yl)methyl benzoate* (**5w**) Yield: 81%; white solid; m.p. 88 °C; ESI-MS *m/z* 298.21 [M + H]^+^; ^1^H-NMR (500 MHz, CDCl_3_) δ 11.86 (br. s, 1H, Ar-OH), 7.99 (dd, *J* = 1.0, 8.0 Hz, 2H), 7.69 (dd, *J* = 1.5, 8.0 Hz, 1H), 7.57 (m, 1H), 7.43 (m, 3H), 7.02 (dd, *J* = 0.5, 8.0 Hz, 1H), 6.90 (m, 1H), 4.79 (m, 1H), 4.57 (m, 2H), 4.46 (dd, *J* = 4.5, 9.5 Hz, 1H), 4.40 (dd, *J* = 7.0, 8.5 Hz, 1H); ^13^C-NMR (125 MHz, CDCl_3_) δ 166.87, 166.32, 159.99, 133.77, 133.25, 129.68, 129.64, 128.46, 128.18, 118.74, 116.89, 110.30, 99.98, 69.23, 65.92, 64.37.

*(2-(2-Hydroxyphenyl)-4,5-dihydrooxazol-4-yl)methyl 2-methylbenzoate* (**5x**) Yield: 86%; white solid; m.p. 63 °C; ESI-MS *m/z* 312.10 [M + H]^+^; ^1^H-NMR (500 MHz, CDCl_3_) δ 7.86 (dd, *J* = 1.5, 7.5 Hz, 1H), 7.67 (dd, *J* = 1.5, 8.0 Hz, 1H), 7.40 (m, 2H), 7.22 (m, 2H), 7.02 (dd, *J* = 1.0, 7.5 Hz, 1H), 6.90 (m, 1H), 4.78 (m, 1H), 4.56 (m, 2H), 4.46 (dd, *J* = 4.5, 9.5 Hz, 1H), 4.38 (dd, *J* = 7.0, 8.5 Hz, 1H), 2.55 (s, 3H); ^13^C-NMR (125 MHz, CDCl_3_) δ 167.33, 166.81, 159.98, 140.36, 133.74, 132.29, 131.77, 130.76, 129.03, 128.17, 125.80, 118.73, 116.87, 110.34, 69.15, 65.76, 64.39, 21.78.

*(2-(2-Hydroxyphenyl)-4,5-dihydrooxazol-4-yl)methyl 2-nitrobenzoate* (**5y**) Yield: 74%; pale yellow crystal; m.p. 108–109 °C; ESI-MS *m/z* 343.12 [M + H]^+^; ^1^H-NMR (500 MHz, CDCl_3_) δ 11.80 (br. s, 1H, Ar-OH), 7.91 (dd, *J* = 1.5, 7.5 Hz, 1H), 7.73 (dd, *J* = 2.0, 7.5 Hz, 1H), 7.67 (m, 3H), 7.40 (m, 1H), 7.02 (dd, *J* = 1.0, 8.5 Hz, 1H), 6.89 (m, 1H), 4.75 (m, 1H), 4.58 (m, 2H), 4.44 (dd, *J* = 6.5, 11.0 Hz, 1H), 4.34 (dd, *J* = 6.5, 9.0 Hz, 1H); ^13^C-NMR (125 MHz, CDCl_3_) δ 167.07, 165.17, 159.95, 133.79, 133.02, 132.02, 130.07, 128.28, 127.12, 123.94, 118.74, 116.87, 110.30, 69.31, 67.12, 63.91.

*(2-(2-Hydroxyphenyl)-4,5-dihydrooxazol-4-yl)methyl 2-chlorobenzoate* (**5z**) Yield: 69%; white needle crystal; m.p. 91 °C; ESI-MS *m/z* 332.12: 333.10 (1:1), [M + H]^+^; ^1^H-NMR (500 MHz, CDCl_3_) δ 11.85 (br. s, 1H, Ar-OH), 7.79 (dd, *J* = 1.0, 7.5 Hz, 1H), 7.67 (dd, *J* = 2.0, 8.0 Hz, 1H), 7.42 (m, 3H), 7.29 (m, 1H), 7.02 (dd, *J* = 0.5, 8.5 Hz, 1H), 6.89 (m, 1H), 4.79 (m, 1H), 4.57 (m, 2H), 4.49 (dd, *J* = 4.5, 9.5 Hz, 1H), 4.42 (dd, *J* = 7.5, 9.0 Hz, 1H); ^13^C-NMR (125 MHz, CDCl_3_) δ 166.92, 165.57, 159.97, 133.79, 133.77, 132.84, 131.66, 131.15, 129.60, 128.19, 126.67, 118.74, 116.88, 110.33, 69.19, 66.37, 64.21.

#### 3.1.3. Synthesis of **6a**–**6o**

A solution of compound **4a** (193 mg, 1.0 mmol) and halides (1.1 mmol), NaH (192 mg, 8.0 mmol) in anhydrous THF (8 mL) is heated to reflux for 6 h. After cooling, the solution is quenched with water (95.0 μL, 8.0 mmol), concentrated under reduced pressure until approximately 5 mL remained, and diluted with CH_2_Cl_2_ (50 mL). Then the mixture is separated with CH_2_Cl_2_ (30 mL × 3) and subsequently washed with an aqueous saturated solution of NH_4_Cl (30 mL × 2), brine (30 mL × 3), and dried over anhydrous Na_2_SO_4_. The solvent is concentrated under reduced pressure, and purified by flash column chromatography using petroleum ether/ethyl acetate (12:1, *v*/*v*) as the eluent.

*2-(4-(Methoxymethyl)-4,5-dihydrooxazol-2-yl)phenol* (**6a**) Yield: 77%; pale yellow oil; ESI-MS *m/z* 208.09 [M + H]^+^; ^1^H-NMR (500 MHz, CDCl_3_) δ 7.66 (dd, *J* = 1.5, 8.0 Hz, 1H), 7.39 (m, 1H), 7.01 (dd, *J* = 1.0, 8.5 Hz, 1H), 6.88 (m, 1H), 4.56 (m, 1H), 4.48 (dd, *J* = 8.0, 9.0 Hz, 1H), 4.33 (dd, *J* = 7.5, 8.5 Hz, 1H), 3.67 (dd, *J* = 4.0, 9.5 Hz, 1H), 3.46 (dd, *J* = 6.5, 9.5 Hz, 1H), 3.40 (s, 3H); ^13^C-NMR (125 MHz, CDCl_3_) δ 166.36, 159.92, 137.59, 134.79, 133.46, 129.16, 128.13, 127.90, 118.61, 116.75, 110.60, 73.41, 71.71, 69.98, 65.25, 21.16.

*2-(4-(Ethoxymethyl)-4,5-dihydrooxazol-2-yl)phenol* (**6b**) Yield: 72%; pale yellow oil; ESI-MS *m/z* 222.10 [M + H]^+^; ^1^H-NMR (500 MHz, CDCl_3_) δ 7.71 (dd, *J* = 2.0, 9.0 Hz, 1H), 7.43 (m, 1H), 7.06(dd, *J* = 0.5, 9.0 Hz, 1H), 6.93 (m, 1H), 4.60 (m, 1H), 4.53 (m, 1H), 4.37 (dd, *J* = 7.0, 9.0 Hz, 1H), 3.78 (dd, *J* = 4.5, 9.5 Hz, 1H), 3.64 (m, 2H), 3.51 (dd, *J* = 7.0, 9.5 Hz, 1H), 1.24 (t, *J* = 3.0 Hz, 3H); ^13^C-NMR (125 MHz, CDCl_3_) δ 166.33, 159.93, 133.46, 128.14, 118.63, 116.76, 110.65, 72.45, 70.07, 67.04, 65.29, 15.10.

*2-(4-(Propoxymethyl)-4,5-dihydrooxazol-2-yl)phenol* (**6c**) Yield: 83%; brown oil; ESI-MS *m/z* 236.17 [M + H]^+^; ^1^H-NMR (500 MHz, CDCl_3_) δ 7.66 (dd, *J* = 1.5, 9.0 Hz, 1H), 7.38 (m, 1H), 7.01 (dd, *J* = 0.5, 9.0 Hz, 1H), 6.88 (m, 1H), 4.56 (m, 1H), 4.48 (m, 1H), 4.33 (dd, *J* = 7.0, 8.0 Hz, 1H), 3.72 (dd, *J* = 4.5, 10.0 Hz, 1H), 3.49 (m, 3H), 1.16 (m, 2H), 0.90 (t, *J* = 7.5 Hz, 3H); ^13^C-NMR (125 MHz, CDCl_3_) δ 166.33, 159.94, 133.44, 128.12, 118.61, 116.75, 110.65, 73.39, 72.60, 70.04, 65.30, 22.79, 10.48.

*2-(4-(Butoxymethyl)-4,5-dihydrooxazol-2-yl)phenol* (**6d**) Yield: 79%; yellow oil; ESI-MS *m/z* 250.10 [M + H]^+^; ^1^H-NMR (500 MHz, CDCl_3_) δ 7.66 (dd, *J* = 1.5, 7.5 Hz, 1H), 7.38 (m, 1H), 7.01 (dd, *J* = 0.5, 8.5 Hz, 1H), 6.88 (m, 1H), 4.55 (m, 1H), 4.48 (m, 1H), 4.33 (dd, *J* = 7.0, 8.0 Hz, 1H), 3.72 (dd, *J* = 4.0, 9.5 Hz, 1H), 3.53 (m, 3H), 1.57 (m, 2H), 1.38 (m, 2H), 0.90 (t, *J* = 7.5 Hz, 3H); ^13^C-NMR (125 MHz, CDCl_3_) δ 166.32, 159.94, 133.44, 128.12, 118.61, 116.75, 110.66, 72.65, 71.53, 70.05, 65.30, 31.67, 19.26, 13.86.

*2-(4-((Pentyloxy)methyl)-4,5-dihydrooxazol-2-yl)phenol* (**6e**) Yield: 83%; yellow oil; ESI-MS *m/z* 264.17 [M + H]^+^; ^1^H-NMR (500 MHz, CDCl_3_) δ 7.66 (dd, *J* = 2.0, 9.0 Hz, 1H), 7.38 (m, 1H), 7.01(dd, *J* = 1.0, 8.5 Hz, 1H), 6.88 (m, 1H), 4.55 (m, 1H), 4.48 (m, 1H), 4.33 (dd, *J* = 6.5, 8.0 Hz, 1H), 3.72 (dd, *J* = 4.5, 9.5 Hz, 1H), 3.52 (m, 3H), 1.58 (m, 2H), 1.32 (m, 4H), 0.87 (t, *J* = 7.0 Hz, 3H); ^13^C-NMR (125 MHz, CDCl_3_) δ 166.33, 159.94, 133.44, 128.12, 118.61, 116.75, 110.66, 72.63, 71.86, 70.04, 65.30, 29.28, 28.27, 22.48, 14.00.

**Figure 3:**
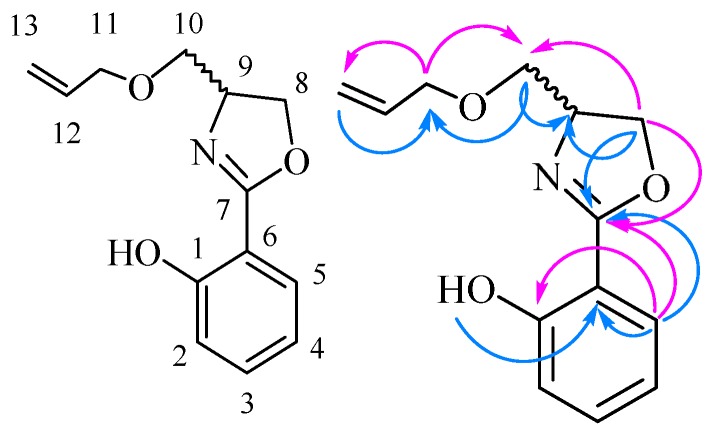
The main HMBC of compound **6f**.

*2-(4-((Allyloxy)methyl)-4,5-dihydrooxazol-2-yl)phenol* (**6f**) Yield: 87%; yellow oil; ESI-MS *m/z* 234.12 [M + H]^+^; ^1^H-NMR (500 MHz, CDCl_3_) δ 11.99 (br. s, 1H, Ar-OH), 7.66 (dd, *J* = 1.5, 6.5 Hz, 1H), 7.38 (m, 1H), 7.01 (dd, *J* = 1.0, 8.5 Hz, 1H), 6.88 (m, 1H), 5.92 (m, 1H), 5.29 (dq, *J* = 1.5, 17.0 Hz, 1H), 5.21 (dq, *J* = 1.5, 10.5 Hz, 1H), 4.57 (m, 1H), 4.49 (m, 1H), 4.35 (dd, *J* = 7.0, 9.0 Hz, 1H), 4.04 (dq, *J* = 1.5, 5.5 Hz, 2H), 3.73 (dd, *J* = 4.5, 9.5 Hz, 1H), 3.49 (dd, *J* = 7.0, 9.5 Hz, 1H); ^13^C-NMR (125 MHz, CDCl_3_) δ 166.39, 159.92, 134.37, 133.48, 128.14, 118.63, 117.42, 116.75, 110.61, 72.46, 71.92, 69.94, 65.25. The structure of **6f** was also confirmed by 2D-NMR data and can be seen in Table 4 and Figure 3.

**Table 4:** HMBC data of compound **6f**.

^13^C-NMR			^1^H-NMR		
C No.	δ_C_	HMBC Correlations	H No.	δ_H_	HMBC Correlations
1-C	159.67, C	Ar-OH, 2-H, 3-H, 5-H	Ar-OH	12.22, s, 1H	1-C, 2-C, 6-C
2-C	116.93, CH	Ar-OH, 4-H	2-H	7.65, dd, *J* = 1.5, 80 Hz, 1H	1-C, 3-C, 7-C
3-C	134.23, CH	2-H, 4-H	3-H	7.49–7.46, m, 1H	1-C, 5-C, 6-C
4-C	119.41, CH	5-H	4-H	6.97–6.94, m, 1H	1-C, 2-C, 3-C, 5-C, 6-C
5-C	128.28, CH	3-H, 4-H	5-H	7.02, dd, *J* = 0.5, 8.5 Hz, 1H	1-C, 4-C, 6-C, 7-C
6-C	110.57, C	Ar-OH, 4-H, 5-H	6-H	-	-
7-C	165.84, C	2-H, 5-H, 8-H, 9-H	7-H	-	-
8-C	71.56, CH_2_	9-H	8-H	4.57–4.54, m, 1H; 4.32–4.30, m, 1H	7-C, 9-C, 10-C
9-C	65.19, CH	8-H, 10-H	9-H	4.62–4.58, m, 1H	7-C, 10-C
10-C	71.80, CH_2_	8-H, 11-H	10-H	3.64–3.57, m, 2H	9-C, 11-C
11-C	69.71, CH_2_	10-H	11-H	4.03–4.02, m, 2H	10-C, 12-C, 13-C
12-C	135.49, CH	11-H, 13-H	12-H	5.93–5.86, m, 1H	10-C
13-C	117.06, CH_2_	11-H	13-H	5.28–5.24, ddd, *J* = 1.5, 3.5, 17.5 Hz, 1H; 5.17–5.15, m, 1H	10-C, 12-C

*2-(4-((Prop-2-yn-1-yloxy)methyl)-4,5-dihydrooxazol-2-yl)phenol* (**6g**) Yield: 69%; yellow oil; ESI-MS *m/z* 232.10; [M + H]^+^; ^1^H-NMR (500 MHz, CDCl_3_) δ 11.98 (br. s, 1H, Ar-OH), 7.66 (dd, *J* = 1.5, 6.5 Hz, 1H), 7.37 (m, 1H), 7.03 (dd, *J* = 1.0, 8.5 Hz, 1H), 6.88 (m, 1H), 4.51 (d, *J* = 4.5 Hz, 2H), 4.49 (m, 1H), 4.11 (s, 2H), 3.73 (dd, *J* = 4.5, 9.5 Hz, 1H), 3.49 (dd, *J* = 7.0, 9.5 Hz, 1H), 3.35 (s, 1H); ^13^C-NMR (125 MHz, CDCl_3_) δ 166.39, 159.92, 134.37, 133.48, 128.14, 118.63, 117.42, 116.75, 110.61, 72.46, 71.92, 69.94, 65.25.

*2-(4-(((4-Chlorobenzyl)oxy)methyl)-4,5-dihydrooxazol-2-yl)phenol* (**6h**) Yield: 77%; pale pink oil; ESI-MS *m/z* 318.09: 320.02 (3:1), [M + H]^+^; ^1^H-NMR (500 MHz, CDCl_3_) δ 7.65 (dd, *J* = 1.5, 8.0 Hz, 1H), 7.39 (m, 1H), 7.31 (dt, *J* = 2.0, 7.5 Hz, 2H), 7.24 (m, 2H), 7.01 (dd, *J* = 0.5, 9.0 Hz, 1H), 6.88 (m, 1H), 4.57(m, 1H), 4.52 (d, *J* = 4.0 Hz, 2H), 4.49 (m, 1H), 4.33 (dd, *J* = 7.0, 8.0 Hz, 1H), 3.73 (dd, *J* = 4.0, 9.5 Hz, 1H), 3.53 (dd, *J* = 6.5, 9.5 Hz, 1H); ^13^C-NMR (125 MHz, CDCl_3_) δ 166.45, 159.91, 136.38, 133.58, 133.54, 128.98, 128.64, 128.15, 118.67, 116.77, 110.53, 72.73, 71.93, 69.73, 65.24.

*2-(4-(((2-Methylbenzyl)oxy)methyl)-4,5-dihydrooxazol-2-yl)phenol* (**6i**) Yield: 88%; pale yellow oil; ESI-MS *m/z* 298.10 [M + H]^+^; ^1^H-NMR (500 MHz, CDCl_3_) δ 7.65 (dd, *J* = 2.0, 9.0 Hz, 1H), 7.38 (m, 1H), 7.29 (m, 1H), 7.21 (m, 3H), 7.01 (dd, *J* = 0.5, 8.0 Hz, 1H), 6.88 (m, 1H), 4.58 (m, 3H), 4.48 (dd, *J* = 8.5, 9.5 Hz, 1H), 4.32 (dd, *J* = 7.0, 8.0 Hz, 1H), 3.77 (dd, *J* = 4.0, 9.5 Hz, 1H), 3.53 (dd, *J* = 6.5, 9.5 Hz, 1H), 2.32 (s, 3H); ^13^C-NMR (125 MHz, CDCl_3_) δ 166.39, 159.93, 136.85, 135.67, 133.47, 130.36, 128.78, 128.13, 128.08, 125.81, 118.62, 116.75, 110.59, 72.07, 72.01, 69.93, 65.25, 18.79.

**Figure 4:**
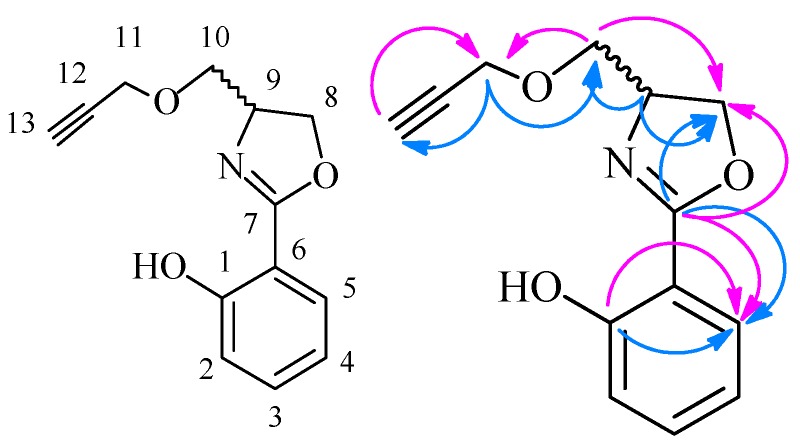
The main HMBC of compound **6j**.

*2-(4-(((2-Fluorobenzyl)oxy)methyl)-4,5-dihydrooxazol-2-yl)phenol* (**6j**) Yield: 63%; pale yellow oil; ESI-MS *m/z* 302.11 [M + H]^+^; ^1^H-NMR (500 MHz, CDCl_3_) δ 11.98 (br. s, 1H, Ar-OH), 7.65 (dd, *J* = 1.5, 6.5 Hz, 1H), 7.38 (m, 2H), 7.29 (m, 1H), 7.14 (m, 1H), 7.06 (m, 1H), 7.01 (dd, *J* = 0.5, 8.0 Hz, 1H), 6.88 (m, 1H), 4.66 (m, 2H), 4.59 (m, 1H), 4.48 (m, 1H), 4.35 (dd, *J* = 7.0, 8.0Hz, 1H), 3.79 (dd, *J* = 4.0, 9.5 Hz, 1H), 3.55 (dd, *J =* 7.0, 9.5 Hz, 1H); ^13^C-NMR (125 MHz, CDCl_3_) δ 166.46, 161.80, 159.94, 133.50, 130.12, 129.65, 128.15, 124.18, 118.63, 116.77, 115.41, 115.24, 110.58, 72.15, 69.89, 67.04, 65.17. The structure of **6j** was also confirmed by 2D NMR data and can be seen in Table 5 and Figure 4.

**Table 5:** HMBC data of compound **6j**.

^13^C-NMR			^1^H-NMR		
C No.	δ_C_	HMBC Correlations	H No.	δ_H_	HMBC Correlations
1-C	159.64, C	2-H, 3-H, 5-H	Ar-OH	12.17, s, 1H	-
2-C	116.93, CH	4-H	2-H	7.65, dd, *J* = 1.5, 8.0 Hz, 1H	1-C, 3-C, 7-C
3-C	134.27, CH	2-H, 4-H	3-H	7.49–7.46, m, 1H	1-C, 5-C
4-C	119.42, CH	5-H	4-H	6.98–6.95, m, 1H	1-C, 2-C, 3-C, 5-C, 6-C
5-C	128.30, CH	3-H, 4-H	5-H	7.02–7.01, m, 1H	1-C, 4-C, 6-C, 7-C
6-C	110.52, C	5-H	6-H	-	-
7-C	165.90, C	2-H, 5-H, 8-H, 9-H	7-H	-	-
8-C	69.59, CH_2_	10-H	8-H	4.58–4.55, m, 1H; 4.31–4.28, m, 1H	7-C, 9-C, 10-C
9-C	64.91, CH	8-H, 10-H	9-H	4.63–4.59, m, 1H	7-C, 10-C
10-C	71.12, CH_2_	8-H, 9-H, 11-H	10-H	3.67–3.66, m, 2H	8-C, 9-C, 11-C
11-C	58.38, CH_2_	10-H, 13-H	11-H	4.23–4.22, m, 2H	10-C, 12-C, 13-C
12-C	80.55, C	11-H	12-H	-	-
13-C	77.95, CH	11-H	13-H	3.49–3.48, m, 1H	11-C

*2-(4-(((4-Fluorobenzyl)oxy)methyl)-4,5-dihydrooxazol-2-yl)phenol* (**6k**) Yield: 67%; yellow oil; ESI-MS *m/z* 302.18 [M + H]^+^; ^1^H-NMR (500 MHz, CDCl_3_) δ 7.65 (dd, *J* = 1.5, 8.0 Hz, 1H), 7.38 (m, 1H), 7.28 (m, 2H), 7.04 (m, 3H), 6.88 (m, 1H), 4.57 (m, 1H), 4.52 (m, 2H), 4.47 (m, 1H), 4.33 (dd, *J* = 7.5, 8.5 Hz, 1H), 3.73 (dd, *J* = 4.5, 5.0 Hz, 1H), 3.52 (dd, *J* = 7.0, 9.5 Hz, 1H); ^13^C-NMR (125 MHz, CDCl_3_) δ 166.44, 163.41, 161.46, 159.92, 133.64, 133.62, 133.53, 129.49, 129.43, 128.15, 118.67, 116.77, 115.43, 115.26, 110.56, 72.82, 71.85, 69.80, 65.24.

*2-(4-(((2,6-Difluorobenzyl)oxy)methyl)-4,5-dihydrooxazol-2-yl)phenol* (**6l**) Yield: 79%; pale yellow oil; ESI-MS *m/z* 320.08 [M + H]^+^; ^1^H-NMR (500 MHz, CDCl_3_) δ 11.96(br. s, 1H, Ar-OH), 7.64 (dd, *J* = 1.5, 8.0 Hz, 1H), 7.38 (m, 1H), 7.31 (m, 1H), 7.00 (dd, *J* = 0.5, 8.0 Hz, 1H), 6.92 (m, 2H), 6.87 (m, 1H), 4.69 (m, 2H), 4.56 (m, 1H), 4.46 (m, 1H), 4.30 (dd, *J* = 7.5, 8.5 Hz, 1H), 3.81 (dd, *J* = 4.0, 9.0 Hz, 1H), 3.51 (dd, *J* = 7.5, 9.5 Hz, 1H); ^13^C-NMR (125 MHz, CDCl_3_) δ 166.47, 162.92, 160.93, 159.91, 133.48, 130.40, 128.14, 118.62, 116.75, 111.45, 111.25, 110.59, 72.09, 70.02, 65.06, 60.60.

*2-(4-(((2,4-Dichlorobenzyl)oxy)methyl)-4,5-dihydrooxazol-2-yl)phenol* (**6m**) Yield: 75%; colourless oil; ESI-MS *m/z* 352.14:354.23:353.12 (9:6:1), [M + H]^+^; ^1^H-NMR (500 MHz, CDCl_3_) δ 7.65 (dd, *J* = 1.5, 8.0 Hz, 1H), 7.39 (m, 1H), 7.35 (m, 1H), 7.01 (m, 1H), 6.88 (m, 2H), 6.82 (m, 1H), 4.61 (m, 2H), 4.56 (m, 1H), 4.48 (m, 1H), 4.33 (dd, *J* = 7.0, 8.0 Hz, 1H); ^13^C-NMR (125 MHz, CDCl_3_) δ 166.49, 159.92, 133.55, 131.11, 131.07, 131.04, 130.99, 128.15, 118.67, 116.78, 111.41, 111.25, 111.22, 110.53, 104.01, 103.81, 103.60, 72.10, 69.76, 66.58, 66.55, 65.16.

*2-(4-(((4-(Trifluoromethyl)benzyl)oxy)methyl)-4,5-dihydrooxazol-2-yl)phenol* (**6n**) Yield: 72%; yellow oil; ESI-MS *m/z* 352.10 [M + H]^+^; ^1^H-NMR (500 MHz, CDCl_3_) δ 7.66 (dd, *J* = 1.5, 9.0 Hz, 1H), 7.60 (d, *J* = 9.0 Hz, 2H), 7.42 (d, *J* = 9.0 Hz, 2H), 7.40 (m, 1H), 7.02 (d, *J* = 8.5 Hz, 1H), 4.65 (dd, *J* = 7.5, 12.5 Hz, 2H), 4.59 (m, 1H), 4.50 (m, 1H), 4.37 (dd, *J* = 7.0, 8.0 Hz, 1H), 3.77 (dd, *J* = 4.5, 9.0 Hz, 1H), 3.59 (dd, *J* = 6.5, 9.5 Hz, 1H); ^13^C-NMR (125 MHz, CDCl_3_) δ 166.53, 159.92, 141.99, 133.59, 128.16, 127.56, 125.44, 125.41, 118.71, 116.79, 110.50, 72.72, 72.19, 69.64, 65.24.

*2-(4-(((4-(Benzyloxy)benzyl)oxy)methyl)-4,5-dihydrooxazol-2-yl)phenol* (**6o**) Yield: 87%; brown oil; ESI-MS *m/z* 390.19; [M + H]^+^; ^1^H-NMR (500 MHz, CDCl_3_) δ 7.65 (dd, *J* = 1.5, 8.0 Hz, 1H), 7.39 (m, 1H), 7.31 (dt, *J* = 2.0, 7.5 Hz, 2H), 7.24 (m, 2H), 7.10 (m, 7H), 5.22 (s, 2H), 4.52 (s, 2H), 4.49 (m, 1H), 4.13 (d, *J* = 6.5 Hz, 2H), 3.73 (dd, *J* = 4.0, 9.5 Hz, 1H), 3.56 (dd, *J* = 6.5, 9.5 Hz, 1H); ^13^C-NMR (125 MHz, CDCl_3_) δ 166.45, 159.91, 136.38, 133.58, 133.54, 128.98, 128.64, 128.15, 126.07, 126.01, 125.34, 123.44, 118.67, 116.77, 110.53, 72.73, 71.93, 70.88, 69.73, 65.24.

#### 3.1.4. Synthesis of **7**

Compound **4a** (193 mg, 1.0 mmol) is dissolved in anhydrous CH_2_Cl_2_ (12 mL), and the system was cooled to −80 °C. DAST (345 mg, 2.0 mmol) was added, and the solution was then stirred at −80 °C for 12 h. Then the reaction was quenched with K_2_CO_3_. After returning to r.t., organic fractions were washed successively with saturated aqueous NaHCO_3_ (30 mL × 2), brine (30 mL × 3), and dried over anhydrous Na_2_SO_4_. Then the solution is concentrated under reduced pressure, and purified by flash column chromatography using ether/ethyl acetate (15:1, *v*/*v*) as the eluent to afford compound **7**.

*2-(4-(Fluoromethyl)-4,5-dihydrooxazol-2-yl)phenol* (**7**) Yield: 68%; white needle crystal; ESI-MS *m/z* 196.10 [M + H]^+^; ^1^H-NMR (500 MHz, CDCl_3_) δ 11.79 (br. s, 1H, Ar-OH), 7.67 (dd, *J* = 2.0, 9.0 Hz, 1H), 7.40 (m, 1H), 7.02 (dd, *J*= 0.5, 9.5 Hz, 1H), 6.89 (m, 1H), 4.66 (m, 1H), 4.60 (m, 1H), 4.53 (m, 2H), 4.41 (dd, *J* = 7.0, 8.5 Hz, 1H); ^13^C-NMR (125 MHz, CDCl_3_) δ 167.12, 159.94, 133.81, 128.26, 118.78, 116.87, 110.30, 84.23, 82.85, 68.38, 65.22.

### 3.2. Biology

#### Minimal Inhibitory Concentration (MIC)

The three kinds of Gram-positive bacteria (*Staphylococcus aureus* 1.89, *Bacillus subtilis* 1.88, *Bacillus cereus* 1.1846) and *Escherichia coli* 1.1636, were purchased from the China General Microbiological Culture Collection Center (CGMCC). *Ralstonia solanacearum* (Gram-negative bacteria) was provided by the College of Plant Protection, Southwest University. *Pseudomonas syringae* pv. *Actinidiae*, *Pseudomonas solanacearum E. F. Smith* (Gram-negative bacteria) were provided by the Plant Pathology Laboratory of Northwest A and F University.

Antibacterial activities were evaluated by the micro-broth dilution method in 96-well culture plates using the Mueller-Hinton broth, according to the National Committee for Clinical Laboratory Standards [20,21]. The tested bacteria were incubated in the Mueller–Hinton broth at 30 °C at 190 rpm for 12 h and the spore concentration was diluted to approximately 1 × 10^5^–1 × 10^6^ CFU/mL with Mueller-Hinton broth. After incubation at 30 °C for 24 h, the MICs were examined. Briefly, bacteria were grown to mid-log phase, diluted with fresh Luria-Bertani culture broth to an optical density of 0.08–0.1 at 600 nm (OD_600_) and diluted again 1:100. This suspension (50 µL) was added to wells in a 96 well microtiter plate (Sarstedt) and 50 µL of compound dissolved in DMSO-water was added to give a final concentration of from 125 to 0.245 μg·mL^−1^ and at most 1‰ DMSO by volume. A DMSO negative control and standard antibiotic positive controls (Ampicillin Sodium, 10 μg·mL^−1^) were included in each plate. All compounds were tested in triplicate for each concentration. Plates were sealed with parafilm and incubated at 37 °C (*Pseudomonas syringae* pv. *actinidiae* and *Pseudomonas solanacearum E. F. Smith* at 28 °C) for 12–16 h. The OD_600_ values for each well were determined with a plate reader (Shimadzu, UV1800, Kyoto, Japan) and the data were standardized to the Luria-Bertani culture broth control wells.

## 4. Conclusions

A novel, efficient, synthetic method was developed for the large-scale preparation of (±)-Yanglingmycin and its analogues. In this way, arylnitriles reacted with serinol under the condition of sodium carbonate in dry methanol to obtain 2-aryl substituent of 4,5-dihydrooxazol analogues with good yields. Series of (±)-Yanglingmycin derivatives were designed and synthesized as potent antibacterial compounds, among which **5b**, **5c**, **5d**, **6g**, and **7** were identified as the most promising candidates with good antibacterial activity, but **4b**–**h** did not possess antibacterial activity at all. This result indicated that the alternation in antibacterial activity of these compounds was dominated by unsaturated bond, electronic interaction, intramolecular hydrogen bond, and stereoscopic effect. Neither electron-drawing nor electron-donating groups could perish the antibacterial activities unless introduction 2-hydroxy to the 2-aryl substituent of 4,5-dihydrooxazol analogues. Further research on the mechanism and toxicology for their bioactivity is ongoing and will be reported in due time.
